# Recent advances in modelling of frost formation for mechanical systems

**DOI:** 10.1098/rsta.2024.0369

**Published:** 2025-07-17

**Authors:** Yong Tao

**Affiliations:** ^1^ Mechanical Engineering, Cleveland State University, Cleveland, OH, USA

**Keywords:** frost formation, heat and mass transfer, refrigerated surfaces

## Abstract

The physics underlying frost and ice formation has been extensively studied over the past few decades, with significant contributions to our understanding of this phenomenon. These insights have primarily been applied to engineering systems with refrigerated surfaces, such as refrigerators, freezers and heat pumps of various sizes. Despite considerable progress, the dynamic and complex mechanisms governing frost and ice formation remain an active area of research, as competing factors continue to challenge predictive accuracy. The increasing interest from stakeholders in reducing energy consumption and carbon footprints in mechanical systems further underscores the importance of advancing modelling and simulation capabilities in this domain. This article critically reviews recent advances in frost and ice formation modelling, with a focus on identifying key research gaps and opportunities for further exploration.

This article is part of the theme issue ‘Heat and mass transfer in frost and ice’.

## 1. Introduction

Research into frost formation on cold surfaces of heat exchangers has been active since the 1940s. It is a very important fundamental research topic because of its unique challenge to understand complex physical phenomena and its profound implications in various engineering design and operation of systems related to energy efficiency, safety and industrial applications. Such applications include heating, ventilation, air conditioning and refrigeration systems, where frost accumulation on heat exchanger surfaces acts as an insulating layer, hindering heat transfer efficiency and increasing energy consumption [1–4]. In the aviation industry, frost and ice accumulation on aircraft surfaces can severely impact flight performance and safety [5,6]. In industrial processing applications, such as cryogenic processes, frost formation can cause blockages and reduce process effectiveness, leading to operational inefficiencies and safety hazards [7–9].

However, significant progress in modelling simultaneous heat and mass transfer within densified frost layers did not emerge until around the 1990s. The advent of such models allowed researchers to predict both the spatial distribution and temporal variation of frost properties beyond the experimental capabilities.

Tao *et al.* [10] developed a mathematical model based on porous media theory to predict the spatial and temporal variations of temperature, frost density and the rate of densification within a frost layer on a flat plate, as well as the time-dependent growth of frost thickness. The model introduced an effective mass diffusivity to describe the internal densification of the frost layer, which was observed to be up to seven times greater than the molecular diffusivity of water vapour in air. This finding has since been revisited in recent studies to explore its implication further. The main assumption was that the mass transfer within the frost layers was predominantly due to water vapour diffusion coupled with desublimation. This work laid a foundation for later studies to explore the transport mechanisms within the frost layer during its growth process.

There were also studies dating back to the 1950s and earlier, which have made contributions to understanding frost behaviour. They were included in several recent reviews in the literature [11–14], which provided comprehensive collections of measurement data, experimental methodologies and useful correlations. These works provided a rich source of data for model validation. For example, Song & Dang [13] conducted a detailed review of frost characteristics and their impact on industrial applications, citing 151 studies. Their review covered experimental techniques for measuring frost layer thickness, including direct methods like digital micrometers, Vernier gauges and scanning probe microscopes (SPM), as well as indirect methods like laser displacement gauges, side-view photo analysis, digital image processing, optical signals and neutron radiography. A mass balance method for frost accumulation based on melted frost after defrosting was also reported.

Their analysis highlighted correlations for frost thermal conductivity and heat/mass transfer coefficients. The review encompassed studies from 1960 to 2010, focusing primarily on cold surface temperatures ranging from −20°C to −5°C, with ambient temperatures of 10−30°C. Only a few studies addressed lower temperatures, down to −40°C.

The following was a list of key research gaps identified by Song & Dang [13]:

— The need for real-time validation of frost models concerning frost layer thickness and density.
— The need to investigate uneven frosting at ultra-low temperatures and during advanced stages of frost melting.
— The lack of generic, widely applicable models and correlations.
— The need for more detailed studies on frosting mechanisms during later stages, when frost density increases rapidly due to melting.

These conclusions remain valid, as subsequent studies since 2018 have not reported significant advancements in addressing these concerns. Specifically, the point of lacking generic and widely applicable models and correlations reflects the unique characteristics of frost growth research that all the models and correlations are applicable to specific ranges of conditions validated through even more limited experimental conditions. As discussed later in the conclusion section of this article, the desire to seek comprehensive, or generic models to meet all the application needs may serve as a question for debate within the research community.

Song & Dang [13] also summarized progress in modelling the transition period between crystal growth and full frost layer development. However, the emphasis of their review remained on experimental studies and resulting empirical correlations. As discussed in subsequent sections of this paper, these experimental studies provide a rich database for contemporary analysis using machine learning techniques. Li & Wang [15] presented a communication focusing on predictive modelling of early-stage water droplet freezing, primarily addressing surface conditions such as texture, surface tension, roughness and curvature, as seen in applications like aircraft icing. The review by Leoni *et al.* [11] echoed similar conclusions and emphasized the need for additional experimental data and improved models, particularly to predict frost formation on more complex geometries, such as vertical and parallel plates.

### (a) Scope of this review

This review focuses on advances made over the last three decades in modelling methodologies of frost growth, specifically in applications related to heat and mass transfer and energy consumption in mechanical systems such as refrigeration, heat pumps and freezers. For many related studies in experimental works that were often quoted in the frost modelling literature, but not discussed in this article, readers can refer to the references [11–14] to fill the gap.

The article is organized into the following three main categories:

(1) *Physics-based models* with the basic configuration which is frost formation on a single cold plate exposed to quiescent or parallel moist air flow. These models can be further identified as
  — Macroscale studies: geometrically lumped models (pseudo-0D and 1D models) where the frost thickness and density vary only as functions of time:
    (1.1) $$\delta_{f}=f(t),\;\;and\;\;\rho_{f}=f(t)$$
  — Microscale studies: distributed models with one or more spatial dimensions, incorporating porous media theory or droplet solidification. These models represent frost thickness and density as functions of both spatial and temporal variables:

(1.2) $$\delta_{f}=f(t),\;\delta_{f}=f\left(x;t\right),\;\delta_{f}=f\left(x,y;t\right),\;\rho_{f}=f\left(z;t\right),\;\rho_{f}=f\left(x,z;t\right),\;\rho_{f}=f\left(x,y;t\right)$$

(2) *Data-driven models* including various regression-based models and machine learning approaches used to predict frost growth and behaviour.
(3) *Geometric and surface features* with models extending beyond basic configurations to include parallel plates, cylinders, square/annulus fins and heat exchangers, and examine the role of surface treatments on heat transfer surfaces in reducing or delaying frost accumulation.

For each of these categories, representative studies are reviewed to provide an overview of the state-of-the-art research that can lead to meaningful discussions of future research.

## 2. Physics-based models

In the context of this article, *physics-based models* refer to the following conservation laws of physics:

(1) Mass conservation (continuity equation)
(2) Momentum conservation (momentum equations)
(3) Energy conservation (energy equation)

The computational domains for these models can be categorized into two types. One is *frost domain only* models that focus on the frost layer itself, in which the airside boundary condition is typically prescribed as a convective boundary condition with defined parameters such as mean air velocity, temperature, heat and mass transfer coefficient and humidity. In natural conditions, air velocity is zero, and thermal buoyancy can be included in the energy equation. The cold side of the frost layer is assumed to have a constant cold plate temperature. The other is *air–frost full domain* models that incorporate both the air and frost subdomains, enabling coupled simulations. The air subdomain is modelled using standard computational fluid dynamics (CFD) formulations to resolve air velocity, temperature and humidity fields. Air–frost interface requires a coupling condition at the air–frost interface, based on the principles of mass and energy conservation, to link the air subdomain with the frost domain dynamically.

To capture the behaviour of frost, various properties such as effective thermal conductivity and mass diffusivity must be modelled. These properties depend on one or more independent variables and are determined using different approaches, either using *experimental correlations* or *theoretical derivations*. As will be discussed further, the accuracy of modelling results, regardless of their complexity, can be influenced by the choice and accuracy of these correlations.

The basic physics about the frost formation on cold surfaces are generally agreed upon by researchers as water molecules deposition processes: frost forms when moist air comes into contact with a surface that is well below the freezing point of water. As will be discussed in this article, depending on what stage of the cooling process, water molecules may appear as either vapour or supercooled liquid (supersaturated) forms in surrounding air when in contact with the cold surface. The surface condition at a given moment may be clean without frost or covered with prior formation of frost. Under complex interaction of heat, momentum and mass transfer among various phases of water, air and surface deposition characteristics (often described by surface tension and contact angles), water vapour or droplet becomes ice crystal, forming porous structure as a result of desublimation (vapour to solid), solidification (liquid to solid), refreezing (melting and solidification micro cycles) or some combinations. As the frost layer grows to a significant thickness, water deposition further contributes to both the frost thickness growth and frost densification, which make the comparison of experimental works with different modelling results very challenging. Some simulation studies reported without the experimental validation that the interfacial temperature at the frost surfaces was below the freezing temperature of water as discussed below in this article.

### (a) Frost domain only models

**Figure 1.**
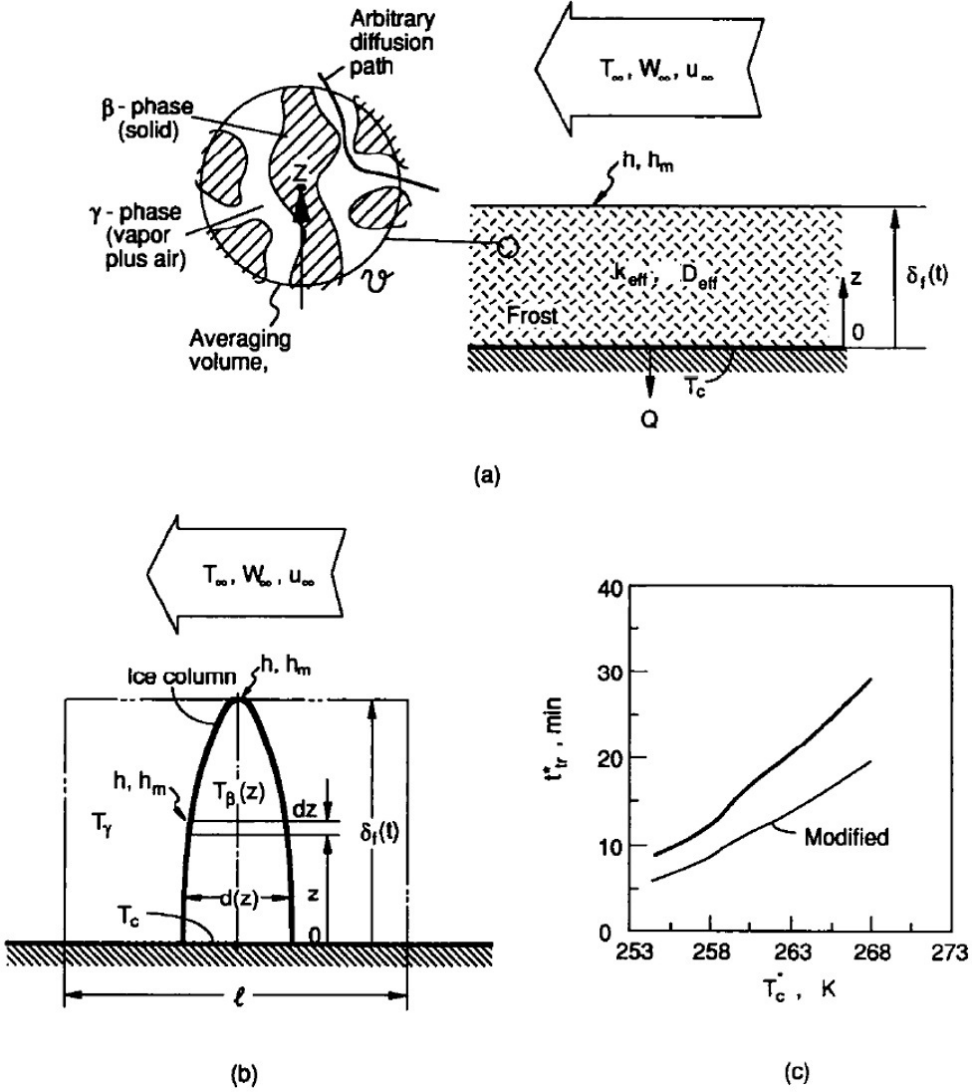
Two-period model predicting frost growth [1]: (a) porous-media model describing thermal and mass diffusion within the frost layer during the full growth period; (b) ice column model for initial growth period; (c) transition time between two periods based on simplified empirical observations: moist air temperature, *T_∞_* , humidity ratio, *W_∞_* , velocity*, U_∞_* , heat and mass transfer coefficients, *h, h_m_*, cold plate temperature, *T*_c_, ice column temperature, *T*_β_ , ice column diameter, *d*, frost thickness, *δ*_f_ , frost density, *ρ*_f,_ frost effective thermal conductivity, *k*_eff_ and effective mass diffusivity, *D*_eff_ . Reprinted from [10], with permission from Elsevier.

Tao *et al*. [10] developed a two-period modelling process, based on experimental data. The test apparatus was designed with a fundamental configuration: controlled ambient air flows in parallel to a controlled cold surface where frost grows. The model applied either natural convection or forced convection boundary conditions on the air side. An initial-stage period featured ice column growth model. The second, full growth period, for the first time, a porous media theory was applied to predict the spatial and temporal variations of temperature, frost density and rate of densification. Figure 1 shows the key variables and parameters that describe the model: moist air temperature, *T_∞_*, humidity ratio, *W_∞_*, velocity*, U_∞_*, heat and mass transfer coefficients, *h* and *h_m_*, cold plate temperature, *T*_c_, ice column temperature, *T*_β_, ice column diameter, *d*, frost thickness, *δ*_f_, frost density, *ρ*_f_, frost effective thermal conductivity, *k*_eff_ and effective mass diffusivity, *D*_eff_ . As a result, the frost layer thickness and the frost density on a flat plate were predicted as a function of growth time, which were compared with the measured values within the acceptable uncertainty. The full-growth model introduced an effective mass diffusivity to describe the internal densification of the frost layer which could be up to seven times larger than the molecular diffusivity of water vapour in air. This observation was later further studied by Bartrons *et al.* [16]. Later, Iragorry *et al*. [1] included the Drop Wise Condensation (DWC) period preceding the Solidified Liquid Tip-Growth (STG) period and the following Densification and Bulk Growth (DBG) period. Leoni *et al.* [11] compare this process with other similar definitions of the overall frost growth process. All the models, for various degrees, cover the detailed discussions on three types of parameters: (i) independent variables, (ii) dependent variables, which have strong application implications, and (iii) theoretical or empirical parameters that are the integral parts of the model linking the independent variables (inputs) to the dependable variables (outputs). Table 1 lists those parameters, including frost thickness correlations from 1970s to 2010s, frost density correlations from 1970s to 2010s, frost thermal conductivity correlations from 1950s to 2010s, heat transfer coefficient correlations from 1950s to 2010s and mass transfer coefficient correlations from 1970s to 2010s in several tables of reference [11].

**Table 1.** Variables and parameters in frost growth models^a^.

independent variables	symbols	units	dimension-less form	uncertainties	sensitivities to dependent variables	representative references^b^
cold surface temperature	*T*_w_	^o^C		low	high	[17]
ambient air temperature	*T*_a_	^o^C		low	high	[1,18]
frosting time	*t*	s, min or h	Fo	low	high	[1,18]
air humidity ratio	*W*	—		medium	medium	[1,18]
air velocity	*u*	m s^−1^	Re	medium	high	[1,18]
heat transfer coefficient	*h*_f_	W m^−2^K^−2^	Nu	medium		[1,18]
mass transfer coefficient	*h*_m_	m s^−1^	Sh	medium	medium	[1,18]
droplet/ice particle size (early stage)	*d*	m		high	high	[1,18]
water droplet contact angle (early stage)	*θ*	degree/radian		medium	medium	[14]
**properties**						
effective thermal conductivity (frost)		W m^−1^K^−1^		high	high	
effective mass diffusivity (frost)	*D*_eff_	m^−2^/s^−1^		high	high	
Prandtl number	*Pr*			low	low	
**dependent variables**						
frost thickness	*δ*_f_	m	*δ*_f_/*L*	medium		
frost density	*ρ*_f_	Kg m^−3^	*ρ*_f_/*ρ*_i_	high		

^a^ Please see the reference [2] for the following detailed tables in [2]: Table 4 Frost thickness correlations from 1970s to 2010s. Table 5: Frost density correlations from 1970s to 2010s. Table 6: Frost thermal conductivity correlations from 1950s to 2010s. Table 9: Heat transfer coefficient correlations from 1950s to 2010s. Table 10: Mass transfer coefficient correlations from 1970s to 2010s.

^b^ May use different symbols.

#### (i) Early growth period

This period is defined based on the observation in experiments. The cooling surface is initially subject to a moist air ambient temperature above freezing temperature. Condensation then occurs when the moist air reaches below the freezing temperature. Those studies that used the experimental observations to arrive at quantitative correlations among the key parameters were mainly under a quiescent ambient condition (natural convection). Zhao *et al.* [12] presented a recent review on the fundamentals of phase changes associated with droplet freezing and the significance of cooling surface characteristics.

The early growth period is relatively short compared with the overall impact of frost growth to heat and mass transfer by cooling surface, as discussed by Tao *et al.* [10]. In fact, the droplet formation on the cooling surface enhances the heat transfer. However, the quantification of condensing and freezing droplets plays an important role by providing the transition condition to the bulk frost layer model description [10]. In [10], an ice column growth model was proposed (see figure 1b). This model assumed a one-dimensional growth of an ice column in the vertical direction, *z*, through the deposition of desublimated water vapour on to the ice column surface. As a result, the diameter of ice column varied with *z* and *t,* along with the column height.

Xu and Wang [19] applied a two-dimensional cellular automata model to simulate the early-stage frost formation process on a cold flat plate, which could capture the main fractal growth characteristics of the frost crystals and analyse the variations of physical properties like frost thickness, porosity and effective thermal conductivity over time, as well as the influences of initial nuclei distribution and supersaturation. This method has the capability to simulate a fractal-like crystal tree growth pattern resembling the real frost microstructure. The two-dimensional frost micro-scale geometry allows the determination of such frost properties as frost density and thermal conductivity with the aid of empirical correlations. The model, however, could not be extended to full frost growth period. It assumes the frost layer is a homogeneous porous medium at the early stage, which is overly simplified. While the model focuses on frosting due to supersaturation (i.e. solidification of supercooled liquid), it could be easily applied to the desublimation process where water vapour in the air stream is changed to ice.

#### (ii) Full frost growth period

Hermes [20] presented an algebraic first-principles simulation model based on macroscopic heat and mass balances in the frost layer. Formulation of the model used a dimensionless approach and analytical solution to obtain an expression for the time evolution of frost thickness:

(2.1) $$X^{2}+d_{1}X-d_{0}\tau =0$$

which yields a positive root, *X*, the dimensionless frost thickness with the plate length as the reference length:


(2.2) $$X=\frac{\sqrt{d_{1}^{2}+4d_{0}\tau }-d_{1}^{,}}{2}$$

where *X* is, through the coefficients of *d*_0_ and *d*_1_, a function of dimensionless time, *τ*, supersaturation degree related to humidity ratio, air–frost temperature difference and Nusselt number. *τ* is defined as $k_{f}t/p_{f}c_{p}L^{2}$. This is a lumped frost model in which all the dependent variables do not vary with the frost layer, and they are only the functions of time. The model results compared well with experimental data within the ranges of supersaturation degree (0.0057−0.0090), dimensionless temperature (5.3–8.5) and Nu (29.3–40.6), which corresponded to the experimental relative humidities of 50% and 65%, cold surface temperature of −9 and −5°C, air temperature of 22°C and air velocity of 0.7 m/s^-1^. This functional relation was consistent with many studies [21–25]. Wang *et al.* [26] conducted a similar, lumped model that is applicable across a wide range of frosting conditions, including air temperatures from −8°C to 25°C, cold plate temperatures from −16°C to −4°C, air humidities from 30% to 80%, and air velocities from 0.7 to 5 m/s^-1^.

Negrelli *et al.* [27] used a modified Diffusion-Limited Aggregation (DLA) method to model the frost growth and densification processes. The DLA model was originally developed to create fractal geometry simulating the molecular or particle diffusion process. Since during the bulk growth period, frost growth is dominated by vapour desublimation through combined heat and mass diffusion, the DLA model can be an effective modelling tool. The model proposed two different particle release patterns to simulate the growth and densification of the frost layer. Calculation of frost mass and porosity was based on the volume of ice particles and the total volume of the frost layer. To find the effective thermal conductivity of frost, a separate discretization of the energy conservation equation, using the finite-volume method, was adopted to solve iteratively using the TDMA algorithm. The important limitation of this method is to define the proper particle numbers to be released in the model. This parameter must be guided by empirical correlations of frost density and thickness. Therefore, the model is not a completely stand-alone solution for evaluating the thermophysical properties of heterogeneous frosted media, particularly the effective thermal conductivity of frost.

### (b) Air–frost full domain models

Due to the wide availability of CFD simulation tools, many studies have been reported in the literature where the coupled domain of moist air, interfacing with cold surface where frost grows, is meshed for full domain computation to solve for frost properties. There are many innovative methods that treat the moving interfacial condition between the frost and air [22–24,28] to achieve the so-called high-fidelity modelling results.

Kim *et al.* [23] present a three-zone numerical model, which defines air, crust and frost as three discretized zones. The crust zone was defined as the interface region separating frost layer and ambient air. This approach created a way to allow the moving frost surface into the predefined computational grids (meshes) that formed the crust zone. A concept of frost formation resistance, *R*, was defined as the resistance to mass transfer during the phase change from water vapour to frost, as appearing as a mass source term within a frost control volume:


(2.3) $${\dot{m}}^{\prime \prime \prime }=\frac{\Delta \Phi }{R}\left[kg/m^{3}\;s\right]$$

*R* was calculated based several variables, including a correction constant, the heat flux through the control volume, the volume and surface area of a control volume (a single grid), and the specific enthalpy of sublimation of water. If all heat loss of the control volume escaped to the cold plate by heat conduction via the frost, the heat flux was then calculated from a correlation containing the effective thermal conductivity of frost in the control volume, and thermal conductivity of frost adjacent to the cold plate, distinguished by the frost temperature in the control volume or by the cold-plate temperature, and the distance between the control volume and the cold plate. The effective thermal conductivity of frost as a function of frost density, reported in the literature, was used.

For the crust zone, a threshold frost density was defined, using an empirical correlation with an error band about 8%:

(2.4) $$\rho_{f.th}=\left(0.627{\left(1+\frac{T_{p}}{273.15}\right)}^{1}{\left(1+\frac{T_{in}}{273.15}\right)}^{1/3}-0.565\right)\rho_{ice}.$$

Guided by the empirically determined source terms, the finite volume method (SIMPLER package) was applied to all three zones in a two-dimensional mesh covering the parallel flow (*x*) direction and the frost growth (*y*) direction. To non-dimensionalize the frost thickness, the model defined the time-dependent frost thickness as the reference length scale in the *y* direction instead of flow domain height. Initial conditions assumed a zero-thickness frost layer, bypassing the dropwise condensation period, typical in experiments. The results of frost thickness and density variations as a function of time showed good agreement with an existing experimental study, where the frost thicknesses and frost density were measured in 30 min intervals. This approach reflected the general logics in most modelling studies, that was to use empirically determined frost thickness and density with coarse time resolutions to adjust parameters in the model. It was natural that the final simulation results demonstrated the good agreement. The true contribution of this type of modelling work was that the models were able to show the detailed physical phenomena that could not be measured, such as percentage of mass transfer contributed to densification and frost layer growth, respectively, during a frosting process, the air frost interfacial flow details and speculated density transition, etc. Lee *et al.* [22] extended this study to predict the growth of highly porous frost layers, showing a reasonable agreement with the experiments within a 15% error range.

Armengol *et al.* [29] conducted a study using a two-dimensional approach for the growth rate, allowing frost elements to increase in both height and width. A finite volume method was applied to discretize the governing equations of mass, energy and momentum in a two-dimensional domain. The main contribution was to consider an extended domain in the inlet boundary to study frost formation in the leading edge of the plate. The model coupled air and frost subdomains, treating air as an incompressible Newtonian fluid and the frost layer as a porous medium. This method has been used in other studies [27,30]. As mentioned earlier, this type of modelling requires the input of initial frost density, which can be affected by the working conditions, and may not accurately predict frost growth under extreme conditions, such as high inlet air velocity.

Bartrons *et al.* [30] presented another finite volume method to solve the frost growth using a similar dynamic meshing approach, which captured the air–frost interface and assessed the performance of various empirical correlations for frost growth under different experimental conditions. The frost layer was modelled as porous media using the local-volume averaged method. Following a one-dimensional model's results (thickness-wise variation), similar to Tao *et al.* [10], authors applied the method to a two-dimensional simulation, including the air domain meshing, to the same configuration and conditions as an experimental study by Kwon *et al.* [31] with noticeable differences. One of the key observations was the identification of the need for more experimental works to study high-porosity frost layers. Adopting the same approach as in [10], the authors confirmed that mass diffusion resistance factors need to be greater than 1 to accurately capture the frost growth, as lower values tended to underestimate the total frost deposition. The study reached the same conclusion as many studies, that lack of consensus on the best empirical correlations to use for modelling frost formation was a major concern. They also extended their study to a fixed-grid numerical model [16,30], which traced the moving interface between air and frost, while maintaining the numerical grids (mesh size) unchanged. The study concluded that the results were sensitive to the mesh size and further study was needed to optimize its configuration for better accuracy; for example, making the mesh denser.

**Figure 2.**
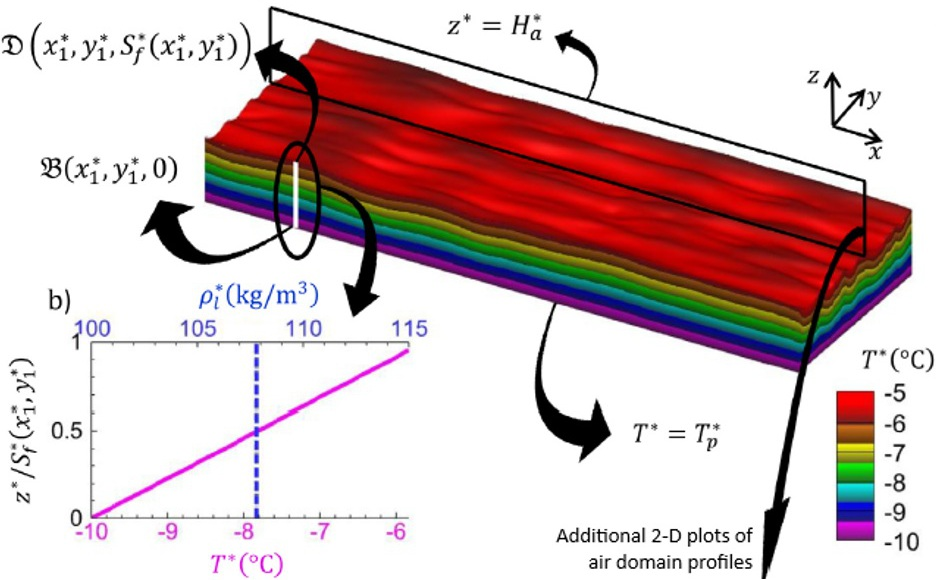
Three-dimensional visualization of the frost layer at an instant in time with contours of temperature subject to turbulent flow [16,25]. The frost layer thickness varies along *x* and *y*. At point D on the frost surface, the thickness is given by *$S_{f}^{*}.$*The plate temperature is held constant at *$T_{p}^{*}$,* but the frost surface temperature varies in *x* and *y*. As shown in panel (b), the temperature increases linearly from the plate temperature to the frost surface temperature. The frost density also varies along *x* and *y*. However, along the *z*-direction, only an average frost density value is considered (i.e. no variation along *z*). Reprinted from [32], with permission from Elsevier.

Farzaneh *et al*. [32] presented a three-dimensional, numerical simulation study of frost formation on a flat plate subjected to turbulent flow as an extension of the study by Zgheib *et al.* [33]. This recent study was perhaps the most complete direct simulation for the physics-based model, although the study was limited to a single Reynolds number of 180. The model used the frost density correlations which might not be directly from the turbulent conditions. The study reported that the Nusselt and Sherwood numbers were largely independent of free-stream and plate conditions when properly scaled, but sensitive to the choice of frost density correlation, suggesting the need for further research to find the best density correlation, and that future work should extract the dependence of the scaled Nusselt and Sherwood numbers on the Reynolds number. A typical visualization of the air–frost domain profiles is illustrated in figure 2.

There were several studies focusing on supersaturation conditions in the air stream (fogging phenomena) when the frost layer grew [17,18,34,35]. One of the representative studies was from Chen *et al.* [17] in which a fix-grid simulation method was applied by coupling the numerical simulation in the air subdomain and analytical analysis in the frost subdomain. The focus was on addressing key issues related to the air–frost interface, supersaturation, and mass transfer in the frost layer. The moving interface between air and frost that resulted from the frost layer growth was determined from the mass diffusion balance equation at the interface. Like many other studies, an effective diffusion coefficient and an additional empirical mass transfer coefficient were introduced in order to count for the unknown enhancement effect at the interface and to match the experimental results. The empirical mass transfer coefficient was found to depend on the air velocity ranging from 0.04 to 0.12 m/s within the limit of their comparison with experimental results.

One challenge of the above-cited high-fidelity modelling of air-frost full domain is the uncertainty in defining a better method to track the moving boundary of the frost surface, which, by definition, is a mushy zone. The mesh sizes around the interface from both the frost subdomain and air subdomain remain a sensitive parameter for convergence and mesh independency. Nevertheless, continued studies in this area will help cross-validate other types of modelling methods and therefore remain valuable.

## 3. Data-driven models

Recently, there has been an increasing number of studies that apply data-driven models, noticeably the machine learning methodology, to determine the primary frost parameters. The main assumption for this type of study is that the existing data of independent and dependent variables from either experimental, analytical or numerical studies are available and accurate within the defined data range and tolerable uncertainties. Mathematical models are all leading to multi-variable correlations that enable the study of influencing conditions for frost growth.

Tahavvor [36] applied artificial neural networks (ANNs) and fractal geometry to model and predict frost thickness and density on a cold flat plate under forced convection and compared the results to experimental data. The simulation covered both the early-growth and full layer growth with the focus on a one-cell fractal geometry generation and used an ANN model with the Levenberg–Marquardt (LM) update rule to train or update the frost parameters. The model inputs were ambient temperature, cold surface temperature, air humidity, Reynolds number and frost growth time from selected experiments by Mao *et al*. [37,38], one of which was the experimental work analyzed by Tao *et al.* [10]. The outputs of the model were frost thickness and frost density. Again, the overall physical analysis was the same as all the studies in the literature. As the authors stated, the LM method was in no way optimal but was just a heuristic. In other words, it was shown to be practical in computational efficiency. The key coefficients adjusted based on the error analysis compared with experimental results were weighing functions. The results and comparison were limited to the cold surface temperature of 258.15 K, ambient air temperature of 278.15 K, the three humidity ratios of 0.003, 0.004 and 0.005, and two Reynolds numbers based on plate lengths of 1000 and 2000. Within these ranges, the overall agreement between the ANN generated results and measured results was within 2% for frost density and 5% for frost thickness.

Moradkhani *et al.* [39] reported several machine learning models to predict frost density and thickness on cryogenic surfaces under forced convection. It also performed sensitivity analysis to determine the most influential factors. Three machine learning based models, GPR (Gaussian process regression), RBF (radial basis function) and MLP (multilayer perceptron network), exhibited high accuracy in predicting frost density and thickness, with the GPR model for frost thickness and RBF model for frost density being the most reliable in comparison with experimental results. As a result, new empirical correlations were developed by the authors showing better accuracy for predicting frost density and thickness under cryogenic conditions than previous models that were not for cryogenic low temperature applications. The model used a total of 249 data points for frost thickness and 54 data points for frost density from previous studies [7,40], with 80% used for training and 20% for testing using the following correlations for the frost thickness and frost density respectively, using five independent variables.


(3.1) $$\frac{\delta_{f}}{L}=f\left(Re_{L},w_{a},Fo,\left|\frac{T_{a}-T_{w}}{T_{f}}\right|,Pr_{a}\right)$$

(3.2) $$\frac{\rho_{f}}{\rho_{a}}=f\left(Re_{L},w_{a},Fo,\left|\frac{T_{a}-T_{w}}{T_{f}}\right|,Pr_{a}\right)$$

Through the ML training and testing, the results were analysed using four error types: average absolute relative error (AARE), relative root mean squared error (RRMSE), coefficient of determination (*R*2), and arithmetic average error (AAE). The following updated correlations for the frost thickness with the GPR model, and frost density with RBF model, are reported:

(3.3) $$\frac{\delta_{f}}{L}=5.09\times 10^{-7}{\left|\frac{T_{a}-T_{w}}{T_{f}}\right|}^{0.79}{w_{a}}^{0.739}{{{Pr}_{a}}^{-38.22}{Re}_{L}}^{0.285}{Fo}^{0.528})$$

(3.4) $$\frac{\rho_{f}}{\rho_{a}}=8.4\times 10^{-16}{\left|\frac{T_{a}-T_{w}}{T_{f}}\right|}^{1.835}w_{a}^{0.879}Pr_{a}^{-125.37}Re_{L}^{0.254}Fo^{0.8}$$

which were valid for the cryogenic conditions under which the cold surface temperatures ranged from −160°C to – 100°C, and the testing time ranges were from 300 to 10 800 s—other conditions were listed in Mao *et al*. [37]. Therefore, the frosting period was in the full-growth period. The above new correlations corresponded to the AAREs 1.06% for frost thickness and 0.83% for frost density, respectively, for all data points used. More than 99% of data predicted by these models were in ± 10% error bounds.

One of the caveats is that the reported agreement was limited to the available experimental data under forced convection conditions. Like other regression correlations developed from various experimental results, they are as good as the experimental conditions dictate.

Moradkhani *et al*. [41] applied the machine learning models, including multilayer perceptron (MLP), Gaussian process regression (GPR), and radial basis function (RBF), to experimental results by Mengjie *et al.* [42]. The models arrived at the thickness and surface roughness of frost layers on horizontal plates under natural convection conditions:

(3.5) $$\frac{\delta_{f}}{L}=-0.1705424+0.0936721\cdot Fo^{0.1872074}\cdot \varphi^{0.1665852}\cdot T_{r}^{0.6176922}$$

(3.6) $$\frac{R_{f}}{L}=0.036+0.507A_{1}+0.582A_{1}^{4}+2675787.11Gr\;A_{1}^{2}+2829447.55Gr\;A_{1}^{3}$$

where the parameter *A*_1_ is defined as follows,

(3.7) $$A_{1}=\left|2.18-0.34\times 0.9988^{\frac{T_{r}Fo}{w_{a}}}+2.15\times 0.9977^{0.197Fo-T_{r}Fo}-9.7T_{r}\right|.$$

In the above two equations, *φ* is relative humidity, *T*_r_ is dimensionless temperature, *Gr* is Grashof number and *w*_a_ is ambient air humidity ratio. GPR, RBF and MLP exhibited good results in predicting frost thickness.

For frost surface roughness, the RBF and GPR models provided precise and similar predictions, while the MLP model had larger deviations. Considering that studies for frost growth under the natural conditions were limited, this type of work could contribute to the better control for applications like small refrigerators and freezers.

Han *et al.* [43] investigated five regression and machine learning techniques to predict frost layer growth on surfaces with different characteristics. They including three traditional regression methods—multiple linear, LASSO (least absolute shrinkage and selection operator), Ridge—and two ML methods, ANN, and SVM (Support Vector Machine). Both forced and natural convection conditions were considered. For all the experimental data included in the study, an outlier exclusion method was applied based on Fox’s work [44]. 78 data points for forced convection and 68 for natural convection were excluded, corresponding to 7% of the total data points. Outlier detection improved the accuracy of the prediction models by up to 2.4% for forced convection and 1.5% for natural convection. The study concluded that the ANN model was the most accurate in predicting frost layer growth, outperforming other regression models. The study also presented the findings of the nonlinear relationship between surface contact angle and frost thickness. The contact angle served as the indicator of surface characteristics on frost growth. It must be pointed out that the surface contact angles only play a role during the early growth period [1] and may not be significant for the entire frosting process.

Abbas *et al.* [45] presented a study on the prediction of frost thickness on a carbon fibre-reinforced polymer (CFRP) composite fin, aiming at potential heat pump applications. Experimentally measured frost thickness data were processed using the ANN approach with Rectified Linear Unit (ReLU) as activation functions. The study limited the scope to natural convection conditions. For the CFRP surface, it is found that the surface temperature had a significant impact on frost thickness, with a 71.5% and 75.9% increase in frost thickness when the surface temperature was decreased from −10°C to −20°C for ambient air temperatures of 5°C and 10°C, respectively. Although this study reflects a preliminary study, where the modified correlation only captures 82% of the data within a 30% error margin, it has potential to further investigate the effects of CFRP surface on frost growth under forced convection conditions.

## 4. Geometric and surface characterizations

The majority of studies have focused on the basic configuration of frost growth experiments, that is a parallel air flow on a single flat surface, which is maintained at a controlled below-liquid water freezing temperature at atmospheric pressure. This basic configuration is necessary to investigate the physics of frost formation process and underlining heat and mass transfer characteristics for comparison among the models against the benchmark experimental data. From the point of view of engineering design for cooling applications, the understanding of deviation of model prediction is equally, if not more, important at a device or system level. The most significant application is the finned cooling surface in air-refrigerant heat exchangers and microchannel heat exchangers, where the air flow between parallel plates is modelled.

### (a) Geometric configuration

#### (i) Parallel cold plates

Ismail & Salinas [46] presented an early approach of extending the prediction model for the basic configuration of the frost formation process, which was validated experimentally, to the case of flow between two parallel cold plates. It adopted the two-stage model described by Tao *et al.* [10]. In doing so, the authors had to adjust arbitrarily some important input parameters such as initial ice crystal radius and mass diffusivity, using a similar approach as in Tao *et al.* [10]. The main assumption was that the flow field and frost growth were symmetrical around the centreline of the parallel flow. Therefore, the key geometric parameter was the distance from the centreline to the frosting surface. The results showed the differences of frost thickness between single plate and parallel plates were not significant, while differences in frost density were very apparent. Armengol *et al.* [29] conducted a two-dimensional, air-frost full domain simulation for the parallel plates. The physics of the model was essentially the same as that in Na *et al.* [47]. The results showed that the frost thickness was nearly uniform along the flow direction with a slight decrease in frost thickness downstream of the airflow. The team did not present any validation results against an experiment.

About 6 years earlier, Lüer & Beer [48] published their detailed experimental results for the parallel plate configuration and the results from the physics-based model. The measured frost thickness profile did illustrate the near uniform pattern in the centre portion of flow direction and slight decrease in frost thickness in the tailing part (downstream) of the test section. This was consistent with the model prediction by Armengol *et al.* [29] with approximately the comparative temperature ranges: the study in [48] had a cold surface temperature from −6°C to −16 °C and air temperature of 20°C, while the study in [29] indicated the cold surface temperature from −20.5°C to −13.7 °C and air temperature of 19.8°C.

**Figure 3.**
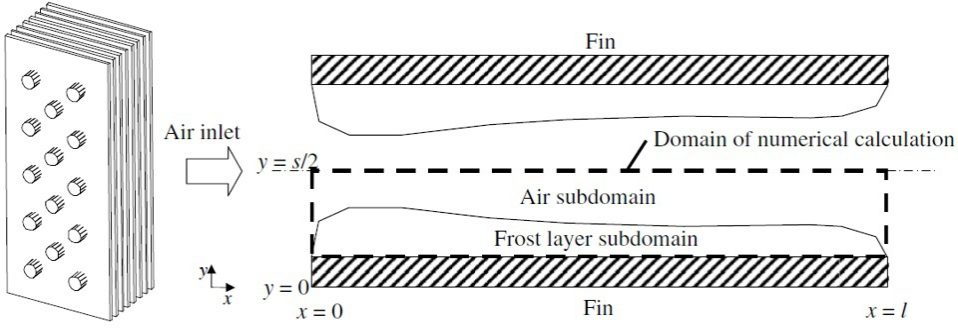
Frost growth on two parallel cold plates forming a channel airflow is modelled as in a symmetric domain where the airflow cross-section is subject to a finite height and an adiabatic thermal boundary condition at the top [42]. Reprinted from [3], with permission from Elsevier.

Another study reported by Lenic *et al.* [3] also conducted a two-dimensional air–frost full domain simulation of frost formation between two parallel plates, although their intention was to model frost formation on a fin-and-tube heat exchanger. The study only reported the frost growth results under one fin space of 1 cm (*S/*2 = 0.5 cm for the computational domain in figure 3). The study shows similar trends of frost thickness distribution and time variation to the study by Lüer & Beer [48].

Coulombe *et al.* [49] presented a pseudo-two-dimensional numerical model to predict frost growth along the length of parallel plates and its effect on the heat recovery efficiency of a counterflow plate heat exchanger under cold climate conditions. The method started with a lumped analysis also called a pseudo-one-dimensional model by applying the surface energy balance principle to the frost layer at a given time to derive the frost thickness. As a new time step progresses, the frost thickness was updated as a function of time. This scheme required the update of frost effective thermal conductivity, heat and mass transfer coefficients from air, and other input variables from the known correlations. The pseudo-one-dimensional model results compared reasonably with the original experimental results of base configuration (single plate). It then extended to the distance step along the flow direction (pseudo-two-dimensional prediction). The important contribution of this study was that the effects of fin spacing were investigated. It was found that larger plate spacing in heat exchangers were less affected by frost growth, as the airflow reduction was lower compared with smaller plate spacing. However, no specific, quantitative conclusions could be drawn, partly because there was no direct experimental validation for the conclusions from the pseudo-two-dimensional model. From their numerical results, one may observe that the average frost thickness after 25 min of frost accumulation remains almost unchanged for the ratio of frost thickness over half of fin spacing less than 0.53 (larger spacing). At the ratio of 0.56, the smallest spacing of 2.5 mm studied shows a noticeable reduction in the average frost thickness and significant push back of frost formation front, the location facing the coming airflow where no frost forms. Also, it was reported that the frost layer was characterized by high porosity (>0.91) regardless of the plate spacing.

The above-discussed studies indicate the lack of a detailed, reliable investigation of the effect of fin spacing on the flow resistance and heat transfer reductions. This effect is well known but the details on the reasons could be studied through the more robust modelling and experimental results.

#### (ii) Cylinder and fin-surfaces in crossflow

Mago & Sherif [35] conducted a semi-empirical, quasi-steady state calculation procedure to predict frost formation and heat transfer on a cylinder surface exposed to supersaturated air with experimental validation. This configuration was limited to the bare tube surface when the adjacent fins commonly co-existed. The model was a physics-based model using a semi-empirical, quasi-steady state approach by considering small time intervals. Empirical correlations for heat transfer coefficient and frost properties were used in the physics model. The results were presented in terms of computed frost formation and heat transfer rate as functions of time and angular position on the cylinder surface. The study was limited to a simple cylinder geometry and therefore called for studies with more complex geometries like finned coils. The model also neglected the angular dependence of some key variables. One of the important findings was that supersaturated air leads to significantly higher frost deposition on the cylinder surface compared with subsaturated air, especially at higher Reynolds numbers.

**Figure 4.**
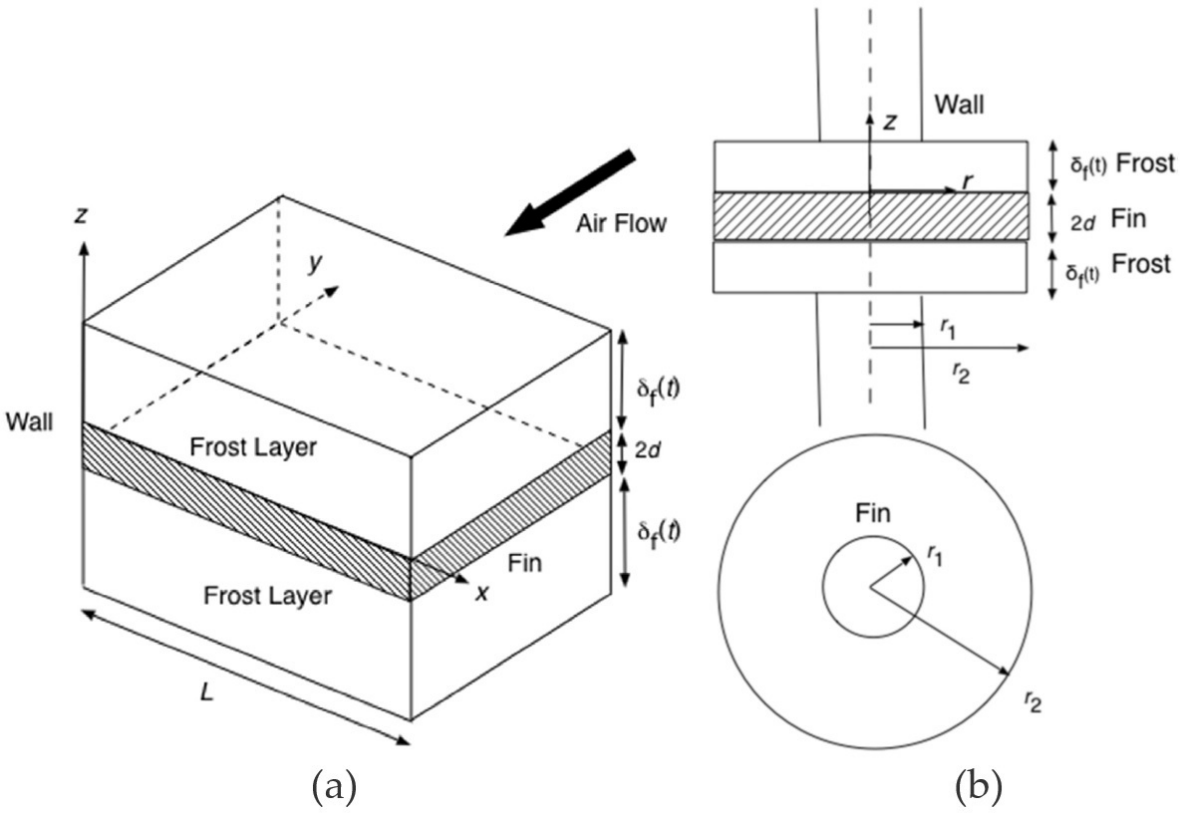
Definition of (a) a square fin and (b) an annular fin, both subject to a cross flow of moist air [44]. Reprinted from [2], with permission from Elsevier.

Benítez *et al.* [2] furthered the study of frost formation over rectangular and annular fins. The study focuses on the frost domain only with prescribed air-side boundary conditions. A new method of normalizing the vertical coordinate of frost domain with the time-dependent frost thickness, the so-called front-fixing method, was developed so that the moving boundary is removed. The closed-loop initial value problem could then be solved analytically using the orthogonal collocation method to discretize the spatial derivatives. The solution was obtained through MATLAB’s ODE solver. Both rectangular and annular fin geometries were analysed (figure 4).

The study showed the following limitations:

— It used a small number of collocation points (*n* = 2) for illustration, though the model predictions were still close to experimental results.
— Further study was needed to properly estimate the super saturation degree at the frost–air interface, which was required to accurately predict frost growth.
— There was increased complexity of modelling annular fins compared with rectangular fins due to the variable cross-sectional area for heat transfer.

Additional experimental studies focused on cylindrical surfaces can be found in the literature [50–52]. Kim *et al.* [50] presented an experimental study of frosting on a cold cylinder surface with the test conditions in Reynolds number range of 700−3000 (air velocities of 0.5−2.0 m/s^-1^), Fourier number of 56.8−295.7 (operating time of 0−100 min), absolute humidity of 0.00280−0.00568 kg/kga, air temperatures of 3−9°C and cold cylinder surface temperatures of −32 to −20°C. The radial variations of frost thickness, frost density and frosting temperature were presented. The radially averaged frost correlations (frost thickness, density, frosting temperature and Nusselt number) agreed with the experimental data within an error of 15%. Barzanoni *et al.* [51] reported another experimental study with the similar experimental conditions and reported the average frost property correlations. Their results confirmed those of Mago & Sherif [35]. Tahavvor & Yaghoubi [52] conducted an experimental study under natural convection conditions. The measurements included heat flux measurement similar to previous studies by Mao *et al.* [37]. A quasi-one-dimensional model was applied to compare with the experimental results with consistent over-prediction of heat flux during the majority of the test period except near the end period of the experiments. These studies provide some limited data points that can be used further to compare different modelling results for the frost growth on cylinders.

### (b) Effects of cooling surface treatments on frost growth

There are several recent papers that focus on modifying surface characteristics such as surface roughness, contact angles, nucleation sites, different materials, etc., in order to reduce or deter frost growth. The main impact of a surface condition is at the early stage of frosting, which is the important consideration in the transition from defrosting and refrosting.

**Figure 5.**
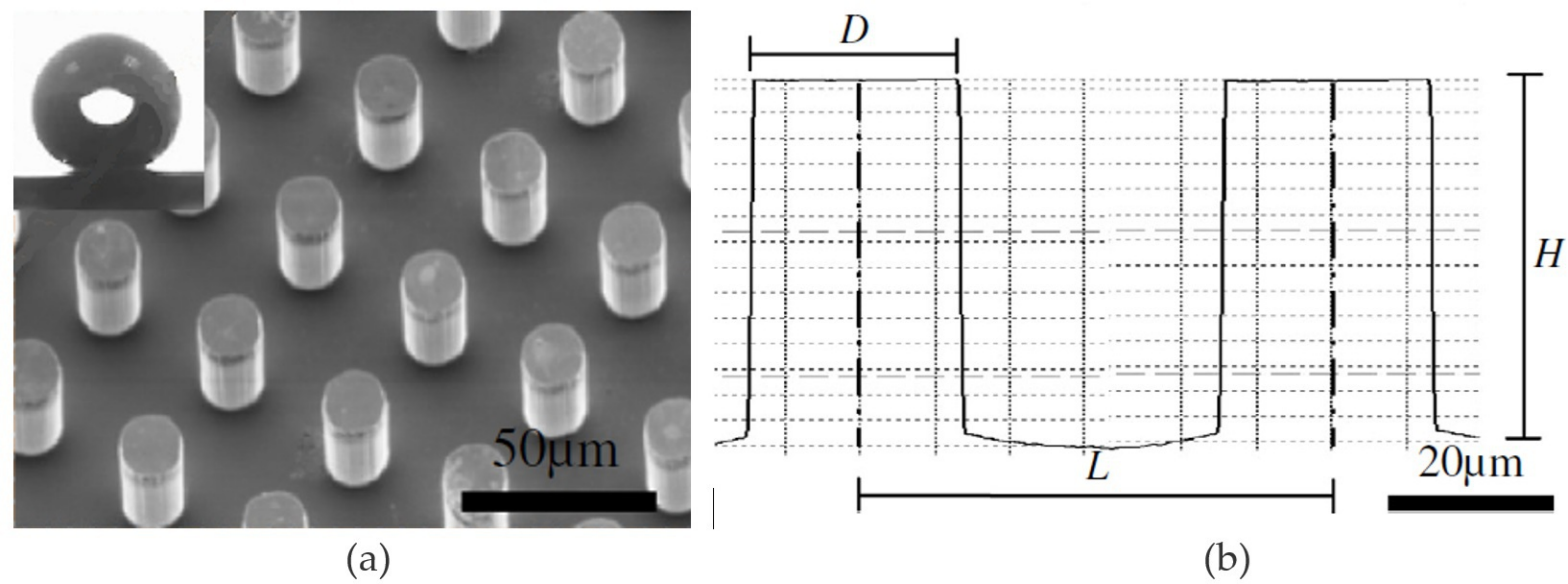
(a) A representative morphology of a micropillar patterned substrate via SEM and a sessile DI water droplet placed on such a substrate, indicating superhydrophobic characteristic [48,53]. (b) Definition of pitch *L*. Reprinted from [53], with permission from Elsevier.

Zhao *et al.* [53] investigated the effects of pillar pitch on the interdroplet freezing wave propagation and ice coverage on *micropillar patterned superhydrophobic surfaces* and proposed three distinctive physical mechanisms/modes to explain the observed phenomena. The test sample of 2 × 2 cm^2^ with an unpatterned margin of 2 mm was created on micropillar patterned superhydrophobic silicon substrates using the standard UV photolithography method, followed by the standard deep reactive ionic etching method. Additional steps were taken to modify the interfacial energy of the fabricated silicon substrates to promote hydrophobicity via chemical vapour deposition. Figure 5 shows the sample.

The experimental setup was designed for condensation and solidification under a natural convection environment. Experiments stopped after the condensing water droplets freeze. No frost growth patterns had been studied. The authors observed three droplet distribution patterns corresponding to three different values of relative pillar pitch (*L*/*D*). Two pillar diameters of 25 and 50 microns were studied. It was found that a relatively small pitch (*L*/*D* = 1.5) showed experimental water bridges from coalesced side droplets, with no ice on the base surface. A moderate pitch (*L*/*D* = 4) showed experimental ice bridges interconnecting pillar to pillar, and the relatively large pitch (*L*/*D* = 7) showed ice bridges primarily along the substrate base surface. Additional discussion on the droplet freezing propagation velocity as a function of *L/D* shed some light on an interesting observation that for each pillar diameter there existed a minimum freezing propagation velocity somewhere around the value of spacing to pillar diameter ratio between 1 and 3 (corresponding to *L*/*D* = 2 and 4.). Understandably, further increase in *L*/*D* would lead the freezing propagation wave to approach the value close to that for a smooth surface. Due to the nature of the randomness of distribution of condensing droplets, the inherited uncertainty in characterizing freezing front propagation (wave) made the reported results non-conclusive. Further study may consider statistical analysis of droplet size distribution and average volume fraction, which would explain the potential delay of freezing when pillars are closer to each other. The height of the pillars is another geometric parameter when the droplets at the pillar tops freeze before those on the substrate base freeze. Within the limits of this study, one can see a significant delay of freezing at the optimal *L*/*D*. Nevertheless, this time difference from the delay of complete droplet freezing is within 3 min. The question would arise as to whether such a time difference could lead to significant impact on the engineering design of a frosting heat exchanger, in which the full growth of frost layer lasts hours before a defrosting cycle kicks in.

**Figure 6.**
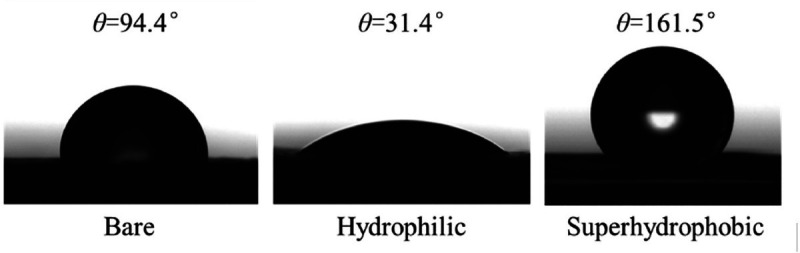
Three surfaces with different contact angles [49]. Reprinted from Applied Thermal Engineering [54], with permission from Elsevier.

Li *et al.* [54] presented a system-level study for an application of *vertical-fin microchannel* frosting evaporator (VMFE). Three different surface conditions were considered (figure 6). It was shown that superhydrophobic coatings on VMFEs resulted in faster defrosting times (about 10% shorter for the condition tested) and better frost growth retardation compared with hydrophilic coatings. Both hydrophilic and superhydrophobic VMFEs showed good cyclic repeatability without capacity attenuation, though the superhydrophobic VMFE had a lower peak capacity but higher average capacity. The vertical fin design with leading edge extensions and gaps between fins formed a ‘transverse-vertical connective drainage channel’ that greatly enhanced the drainage performance of the VMFE. From the photos reported, the author of this review article commented that the reason for the superhydrophobic coatings on a vertical frosting surface was that the frost grain seemed coarser than frost formed on the surface with hydrophilic coating. It would be interesting to investigate more detailed characteristics of microscopic structure of frost layers and the corresponding properties such as density, thermal conductivities, etc.

**Figure 7.**
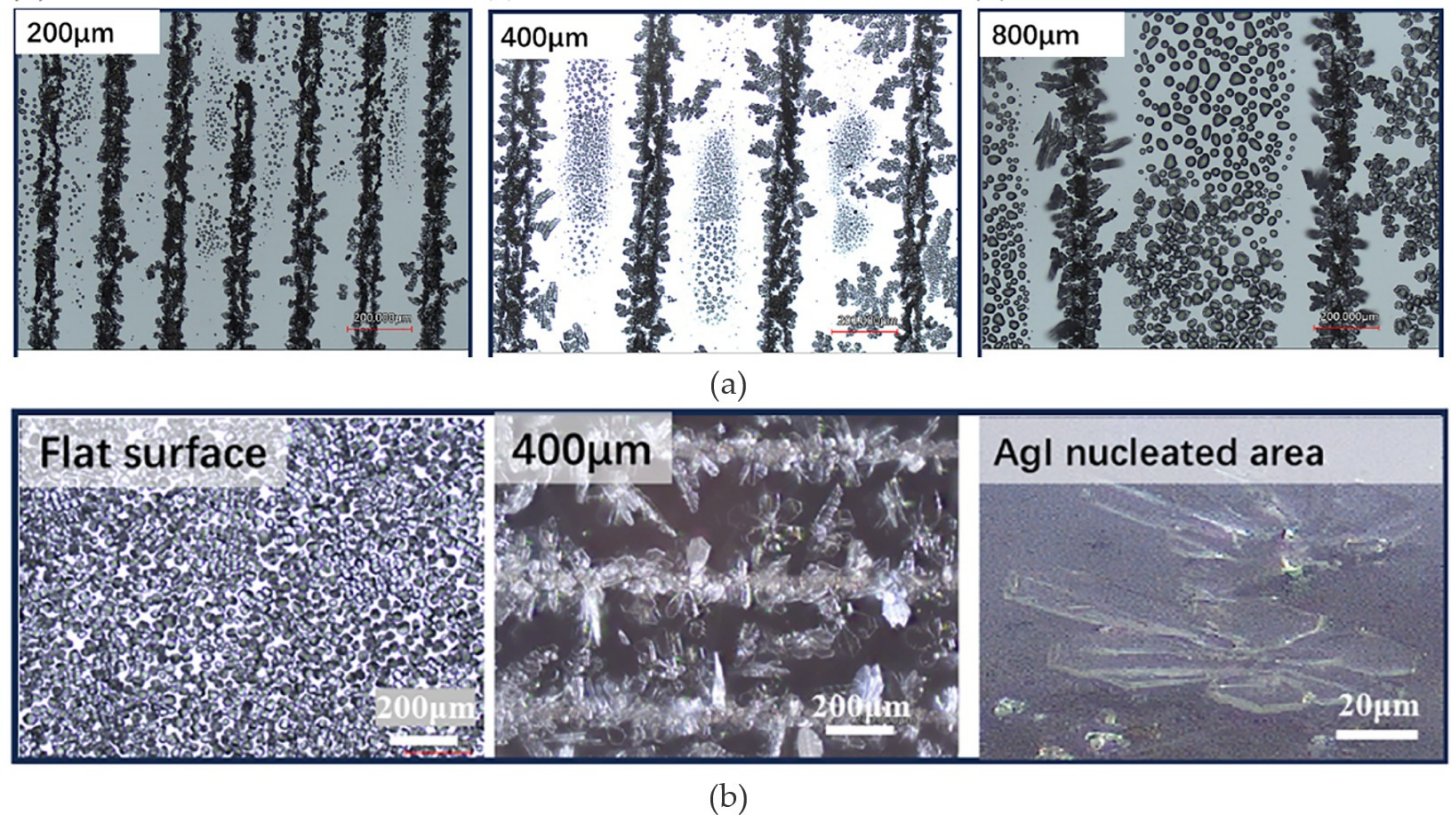
Frost growth on an AgI stripe patterned surface with (a) three different spacings, and (b) with a comparison between a flat surface and a patterned surface (400 micron metre spacing), showing a horizontal growth trend on strip patterned surface [50]. Reprinted from International Journal of Heat and Mass Transfer [55], with permission from Elsevier.

Tang *et al.* [55] investigated the use of *silver iodide (AgI) patterns* to control initial liquid droplet formation and subsequent frost growth under condensation frosting conditions. Frost preferentially initiated from the AgI striped patterns and showed horizontal crystal growth, eventually leading to the formation of frost-free zones in between the stripes. A photolithography method by Okabe *et al.* [56] is applied to fabricate the AgI stripe patterns. Through the fabrication process, three patterns were created on the silicon (SI) substrate with different spacing between the strips (figure 7). The completed samples were then mounted on a copper plate for frost growth testing under natural convection conditions. The frosting patterns revealed horizontal growth of the crystal (figure 7*b*).

To quantify the ice crystals, droplets and frost distribution, a machine-learning algorithm, semantic segmentation convolutional neural network (CNN), was applied to image processing. Distinguishing different phases from images provided a precise and efficient way to evaluate frost growth. The main findings of this study included the observation of horizontal frost growth pattern from the AgI stripes due to the nucleation properties of AgI, which led to the formation of frost-free zones between the stripes. The study explains that the formation of the frost-free zones was influenced by the dynamic interaction between water droplet evaporation and de-sublimation on the frost tip. Water droplets evaporate as a result of a vapour pressure difference between the ice front and the water droplets, creating a localized concentration gradient which drives vapour from the liquid droplets to the ice front, as described by the equation below:

(4.1) , $$\delta_{ff}=\xi \frac{1}{2}\left[1+\sin\left(\theta -\frac{\pi }{2}\right)\right]\left(1-\frac{P_{i}}{P_{w}}\right)\frac{T_{a}}{ST_{w}-T_{a}}$$

where *δ*_ff_ is the frost-free length, *P*_i_ and *P*_w_ are the partial pressure of ice and water vapour, respectively, *S* is supersaturation, *T*_a_ and *T*_w_ are air temperature and frost temperature, respectively, and *ξ* is strip hight. Further investigation is needed to evaluate this conclusion under a wide range of operation conditions, such as force convection and defrosting cycles.

## 5. Summary

The representative studies reviewed above reflect the key developments of modelling techniques for predicting frost growth on mechanically controlled freezing surfaces. Despite the seemingly well-known research topic area, the research community is still active with various capacity of experiments to quantify the fundamental frost growth phenomena through modelling and simulation. The main reason is the large number of variables influencing the ultimate frost growth patterns and its properties that make the scale-up prediction (i.e. extrapolation) beyond the defined conditions impossible without the experimental validation. This therefore leads researchers to repeat the experiments for any deviation of new conditions from the previous ones. The following general conclusions may be drawn:

### (a) General agreements

— The basic configuration of frost growth on a single plate subject to a parallel airflow has been well established. The basic physics include water vapour or supercooled liquid (supersaturation) diffusion into the frost layer under corresponding concentration gradients. The coupled heat and mass transfer in the convective air stream (or quiescent ambient) is the driving force of both frost layer growth and densification.
— Regardless of how the model is lumped, one-dimensional or multidimensional, the conclusion is the same: frost thickness increases with higher air velocity, higher relative humidity and lower cold surface temperature. Frost density increases with higher wall temperature and higher air velocity, but the impact of relative humidity is debatable. Predictive methods for frost density are less accurate than those for frost thickness.
— It has been generally agreed that the frost growth process is influenced by the independent variables and characterized by dependent variables as listed in table 1 and shown in equations (3.1) and (3.2). This list reflects all the dataset and correlations developed using machine-learning modelling techniques. For more comprehensive, detailed lists, the reader is referred to the footnote of table 1 for the reference to Song and Dance’s work [13].

### (b) Recommendations

— Frost growth phenomena are highly complex and the characterization of frost in terms of thickness and density impacting the heat transfer effectiveness of the designed surface. The basic configuration model of parallel flow interacting with the frosting surface can be applied to a different surface or ambient condition with additional parameters that need to be quantified.
— The rich experimental and correlation generated data may provide a source for machine learning techniques to apply to the similar applications. There is a need to compile a comprehensive database by collecting experimental data with clearly defined uncertainties, test conditions and applicable ranges of variables. A mechanism to share the data with the research community through a software platform is needed. The research on the data interoperability, scaling up or down, and cross validation and verification is also needed.
— New integrative methodology should be developed for meaningful applications and made available to practitioners or researchers to apply for new design with different geometries such as horizonal growth versus vertical growth of frost. Machine-learning can play an important role in this area.
— New research activities should also focus on defrosting control strategies when refreezing of melt droplets may present a different initial condition for each refreezing cycle.
— It is much simpler and more practical to define an initial frost thickness and density, which can be correlated with the ambient temperature, humidity and air velocity, but their values are guessed and adjusted based on the overall mass balance of frost accumulation over a long period of experiments. This is because time-domain effects of early-stage growth have large uncertainties in terms of making transition to full growth period. The fundamental study in this area can contribute better definition of transition time, initial frost thickness and density for the full growth period.
— In relation to the modelling of the early frost growth period, it is an active area seeking surface treatments to achieve hydrophobic or hydrophilic conditions. It also has implications for contributing to optimal defrosting processes. There is a need to better understand the sensitivity of various microscopic mechanisms [14] contributing to the overall frost layer formation during the early period.
— It is always a big challenge to experimentally evaluate the microscopic transport phenomena internally within the frost layer, that explains frost densification. A breakthrough in this area will alter the foundation of the physics model that relies on effective thermal and mass diffusion assumption within frost, a porous medium. Some recent studies show that under natural convection, there is evidence of melting and refreezing at the surface of ice/frost at both early and later stages [57]. Modelling efforts to include its effect in the frost layer growth have large discrepancy between the prediction and experimental results under forced convection ambient conditions [8]. Therefore, further studies are needed.
— Modelling work for the frosting surface subject to turbulence flow may need further attention as the frost densification may be affected by induced convection within the porous frost layer due to high air velocity. This is especially critical when the airflow is confined in a narrow passage as in finned heat exchanger applications. This penetration layer of velocity profile, i.e. the slip condition at the top of the frost layer, has been modelled for a porous medium [58] but has not been applied to frost growth study.
— In general, the high-fidelity modelling of air–frost full domain faces the uncertainty in defining a better method to track the moving boundary of the frost surface, which by definition is a mushy zone. The computational time is extensive. The mesh sizes around the interface from both the frost subdomain and air subdomain remain a sensitive parameter for convergence and mesh independency. Nevertheless, continued studies in this area will help cross validate other types of modelling methods and therefore remain valuable.

## Data Availability

This article has no additional data.
